# COVID-19: Immunohistochemical Analysis of TGF-β Signaling Pathways in Pulmonary Fibrosis

**DOI:** 10.3390/ijms23010168

**Published:** 2021-12-24

**Authors:** Caroline Busatta Vaz de Paula, Seigo Nagashima, Vanessa Liberalesso, Mariana Collete, Felipe Paes Gomes da Silva, Alessandro Gonçalves Gomes Oricil, Giovanna Silva Barbosa, Guilherme Vieira Cavalcante da Silva, David Batista Wiedmer, Felipe da Silva Dezidério, Lucia Noronha

**Affiliations:** Postgraduate Program of Health Sciences, School of Medicine, Pontifical Catholic University of Paraná, Curitiba 80215-901, Brazil; seigo.nagashima@pucpr.br (S.N.); vane.hpp@gmail.com (V.L.); mari_collete@hotmail.com (M.C.); paes.silva@pucpr.edu.br (F.P.G.d.S.); alessandro.oricil@pucpr.edu.br (A.G.G.O.); gibarbosa1999@gmail.com (G.S.B.); gui99vcs@gmail.com (G.V.C.d.S.); davidbatistawiedmer@gmail.com (D.B.W.); fsdeziderio@hotmail.com (F.d.S.D.); lnno.noronha@gmail.com (L.N.)

**Keywords:** SARS-CoV-2, TGF-β, fibrosis, fibroblast, diffuse alveolar damage

## Abstract

Acute respiratory distress syndrome (ARDS) followed by repair with lung remodeling is observed in COVID-19. These findings can lead to pulmonary terminal fibrosis, a form of irreversible sequelae. There is evidence that TGF-β is intimately involved in the fibrogenic process. When activated, TGF-β promotes the differentiation of fibroblasts into myofibroblasts and regulates the remodeling of the extracellular matrix (ECM). In this sense, the present study evaluated the histopathological features and immunohistochemical biomarkers (ACE-2, AKT-1, Caveolin-1, CD44v6, IL-4, MMP-9, α-SMA, Sphingosine-1, and TGF-β1 tissue expression) involved in the TGF-β1 signaling pathways and pulmonary fibrosis. The study consisted of 24 paraffin lung samples from patients who died of COVID-19 (COVID-19 group), compared to 10 lung samples from patients who died of H1N1pdm09 (H1N1 group) and 11 lung samples from patients who died of different causes, with no lung injury (CONTROL group). In addition to the presence of alveolar septal fibrosis, diffuse alveolar damage (DAD) was found to be significantly increased in the COVID-19 group, associated with a higher density of Collagen I (mature) and III (immature). There was also a significant increase observed in the immunoexpression of tissue biomarkers ACE-2, AKT-1, CD44v6, IL-4, MMP-9, α-SMA, Sphingosine-1, and TGF-β1 in the COVID-19 group. A significantly lower expression of Caveolin-1 was also found in this group. The results suggest the participation of TGF-β pathways in the development process of pulmonary fibrosis. Thus, it would be plausible to consider therapy with TGF-β inhibitors in those patients recovered from COVID-19 to mitigate a possible development of pulmonary fibrosis and its consequences for post-COVID-19 life quality.

## 1. Introduction

COVID-19 fatalities were observed mainly in elderly patients with relevant comorbidities [1], contrasting with H1N1pdm09 (pandemic caused by the respiratory virus Influenza A, subtype H1N1 in 2009), which mainly affected young adults [2]. Although the respiratory viruses responsible for both outbreaks are different in their demographic risks and pathophysiological mechanisms, both can lead to acute respiratory distress syndrome (ARDS), diffuse alveolar damage (DAD), and terminal fibrosis [3,4].

The progression of ARDS from DAD to tissue repair, remodeling and terminal fibrosis seems to be closely related to a cytokine storm and Renin-Angiotensin System (RAS) imbalance [5,6].

The terminal lung fibrosis is induced by activating the M2 macrophages phenotype, resulting from T-helper 2 (Th2) lymphocytes [7]. The functions of Th2 cells are mediated by Interleukin 4 (IL-4) and Interleukin 13 (IL-13), which induce the activation of M2 macrophages leading to fibrosis through the secretion of Growth Transforming Factor-Beta (TGF-β), stimulating fibroblast proliferation and collagen synthesis [7,8].

In a pulmonary viral infection, oxidative stress is evident in epithelial cells. The lung epithelial cell injury and consequent exposure of the alveolar basal membrane led to an accumulation of TGF-β1, which induces the recruitment of fibroblasts and extracellular matrix (ECM) production. In addition, the expression of collagen-induced by Angiotensin II (AngII) relies on TGF-β1, which subsequently increases the ECM deposition [6].

Myofibroblasts, by synthesizing Smooth Muscle Actin (SMA) [9], can promote irreversible contraction, an essential feature of fibrogenesis, in addition to the production of ECM (Collagen I and III and Fibronectin) [6].

CD44 is a protein activated in inflammatory processes being the central receptor of hyaluronic acid (HA) [10]. The complex CD44-HA leads to activation of the Phosphatidylinositol 3 Kinase/Protein Kinase B (PI3K/AKT) pathway, which induces the reduction in cellular apoptosis, increasing the survival of fibroblasts and myofibroblasts [11].

The expression of Metalloproteinase-9 (MMP-9) can significantly impact the development of pulmonary fibrosis [12]. This enzyme is expressed by alveolar epithelial cells, neutrophils, macrophages, and fibroblasts being able to activate TGF-β1, which contributes to the increase in the active TGF-β pool [13].

Severe Acute Respiratory Syndrome Coronavirus 2 (SARS-CoV-2) uses the Angiotensin-Converting Enzyme 2/Transmembrane Protease Serine 2 (ACE-2/TMPRSS2) as a gateway to infect its host [14]. In addition to this main pathway, SARS-CoV-2 can infect human cells by the endosomal route [15]. Recent reports have implicated the ACE-2-rich lipid rafts as responsible for this endocytic process [16]. The lipid rafts, in turn, are enriched by the Caveolin-1 protein (Cav-1), a protein involved in the negative regulation of TGF-β1 through the internalization of TGF-β membrane receptors [17].

Given the above, the present study analyzed twenty-four cases of biopsies of patients who died due to COVID-19, aiming to understand the behavior of the TGF-β1 pathway and the process of pulmonary remodeling in the severe forms of SARS-CoV-2 lung injury. Understanding the mechanism that leads from the COVID-19 disease to cytokine dysregulation could help identify, in the future, efficient forms to minimizing lung fibrosis, the inflammatory terminal phenomenon that causes irreversible architectural distortion and pulmonary dysfunction [18].

## 2. Results

The relevant demographic, clinical, and histopathological analyses of the groups are summarized in Table 1 and Table 2.

When evaluating the histopathological findings (Table 2), we observed a statistically significant increase in the hyaline membrane, hyperplasia of pneumocytes type II, fibrosis, and immature Collagen III (*p* < 0.001; <0.001; 0.0002; <0.001; and 0.0015, respectively) and a statistically significant decrease in mature Collagen I (*p* < 0.0015) in the COVID-19 group compared to the Control group. On the other hand, when the COVID-19 group was compared to the H1N1 group, we observed a statistically significant increase in hyaline membrane, fibrosis, and decreased Collagen I (mature) in the former group (*p* = 0.009; 0.048, and 0.0006, respectively).

The analysis of the relationship between the use of corticosteroids by the COVID-19 patients and the presence of terminal fibrosis in their lung samples revealed no statistically significant results (*p* = 0.718) (Table 3).

**Figure 1:**
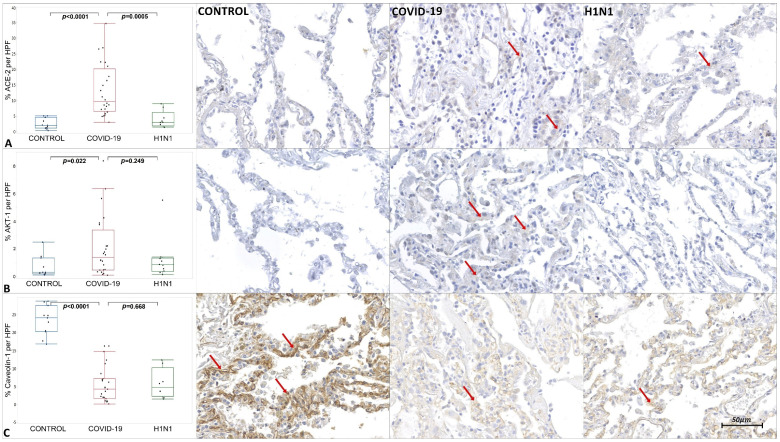
Graphics show tissue immunoexpression of ACE-2, AKT-1 and Caveolin-1 (percentage per HPF). (**A**) Comparative graphs between the CONTROL, COVID-19 and H1N1 groups and photomicrographs of tissue immunoexpression of ACE-2 in type II pneumocytes represented by the red arrow, of the respective groups. (**B**) Comparative graphs between the CONTROL, COVID-19 and H1N1 groups and photomicrographs of tissue immunoexpression of AKT-1 in the alveolar epithelium, represented by the red arrow, of the respective groups. (**C**) Comparative graphs between the CONTROL, COVID-19 and H1N1 groups and photomicrographs of tissue immunoexpression of Caveolin-1 in the alveolar epithelium, represented by the red arrow, of the respective groups.

**Figure 2:**
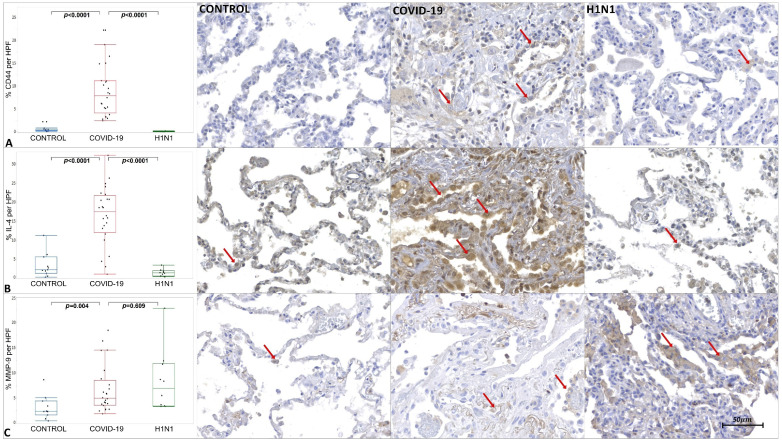
Graphics show tissue immunoexpression of CD44v6, IL-4, and MMP-9 (percentage per HPF). (**A**) Comparative graphs between the CONTROL, COVID-19 and H1N1 groups and photomicrographs of tissue immunoexpression of CD44v6 in alveolar epithelium represented by the red arrow, of the respective groups. (**B**) Comparative graphs between the CONTROL, COVID-19 and H1N1 groups and photomicrographs of tissue immunoexpression of IL-4 in the alveolar epithelium, represented by the red arrow, of the respective groups. (**C**) Comparative graphs between the CONTROL, COVID-19 and H1N1 groups and photomicrographs of tissue immunoexpression of MMP-9 in the alveolar epithelium, represented by the red arrow, of the respective groups.

**Figure 3:**
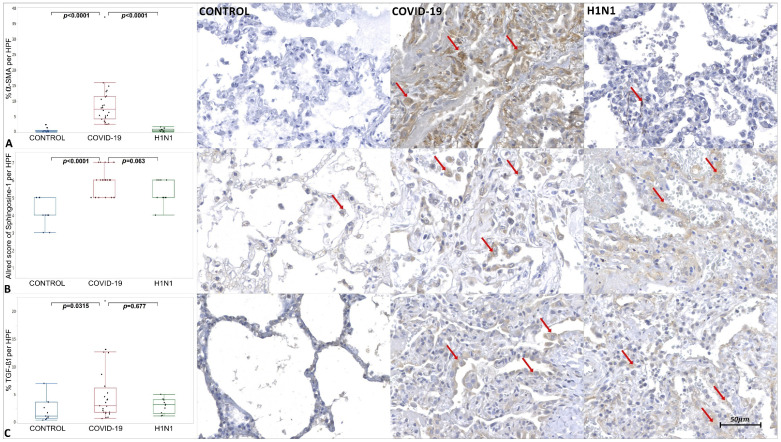
Graphics show tissue immunoexpression of α-SMA and TGF- β1 (percentage per HPF) and Sphingosine-1 (M2 macrophages and type II pneumocytes Allred score). (**A**) Comparative graphs between the CONTROL, COVID-19 and H1N1 groups and photomicrographs of tissue immunoexpression of α-SMA in alveolar epithelium represented by the red arrow, of the respective groups. (**B**) Comparative graphs between the CONTROL, COVID-19 and H1N1 groups and photomicrographs of tissue immunoexpression of Sphingosine-1 M2 macrophages and type II pneumocytes, represented by the red arrow, of the respective groups. (**C**) Comparative graphs between the CONTROL, COVID-19 and H1N1 groups and photomicrographs of tissue immunoexpression of TGF- β1 in the alveolar epithelium, represented by the red arrow, of the respective groups.

The tissue immunoexpression of ACE-2, AKT-1, Cav-1, CD44v6, COX-2, IL-4, MMP-9, SMA, Sphingosine-1, and TGF-β1 in the COVID-19, H1N1, and CONTROL groups are shown in Figure 1, Figure 2 and Figure 3 and Supplementary Table S1.

The COVID-19 samples presented statistically increased tissue immunoexpression of ACE-2 (*p* < 0.0001), AKT-1 (*p* = 0.022), CD44v6 (*p* < 0.0001), IL-4 (*p* < 0.0001), MMP-9 (*p* = 0.004), α-SMA (*p* < 0.0001), Sphingosine-1 (*p* < 0.0001), and TGF-β1 (*p* = 0.0315) when compared to the CONTROL group. We also observed significantly decreased immunoexpression of Cav-1 (*p* < 0.0001) in the COVID-19 compared to the CONTROL group.

When comparing the COVID-19 to the H1N1 group, statistically increased tissue immunoexpression of ACE-2 (*p* = 0.0005), CD44v6 (*p* < 0.0001), IL-4 (*p* < 0.0001), and α-SMA (*p* < 0.0001) were observed in the former. No statistical difference was observed in the tissue expression of AKT-1, Cav-1, MMP-9, Sphingosine-1, and TGF-β1 when testing COVID-19 and H1N1 groups.

## 3. Discussion

### 3.1. Histopathological Findings and Collagens Analysis

The present study’s findings corroborate the literature, since it is observed that COVID-19 patients had an average age of 71.96 years, relevant comorbidities, long periods of hospitalization, and exposure to mechanical ventilation. The age of H1N1 patients was lower (43.5 years) along with the time of hospitalization and mechanical ventilation, validating what was previously seen in other studies [2].

In the histopathological evaluation of this study, DAD was pointed out as the formation of hyaline membranes in the alveolar lumen and hyperplasia of pneumocytes type II, in addition to the presence of terminal fibrosis. Studies report that terminal lung fibrosis is usually present in fatal cases of COVID-19, which is the pathognomonic histopathological aspect of ARDS in the process of repair [19].

SARS-CoV-2 migrates to the lower respiratory tract and mainly affects type II pneumocytes [6], followed by the secretion of cytokines [20]. The inflammation process is followed by alveolar edema and hyaline membranes over the damaged alveolar septa [21]. At the end of this process, septal terminal fibrosis appears, characterized by exacerbated proliferation of fibroblasts and excessive deposition of ECM [21,22]. Collagens I (mature) and III (immature) are the primary components of ECM [23]. Collagen III (immature) seems to predominate at the beginning of the fibrotic process, followed by Collagen I (mature) [24].

The results observed in the COVID-19 patients indicate high levels of Collagen III (immature) compared to the CONTROL group (Table 2). This precursor found in the alveolar compartment is associated with an unfavorable condition and a higher risk of death [25]. Collagen I (mature) was significantly decreased in the COVID-19 group compared to the others (Table 2). Collagen I (mature) is the main structural protein in the pulmonary septal interstitium [26]. It is found in large quantities during chronic pulmonary fibrosis, promoting the destruction of septal architecture and consequent impairment of gas exchange. In addition, the presence of Collagen I (mature) seems to be directly related to the mechanical/biochemical signaling of the actin–myosin contractile system [27].

Type III Collagen is considered an immature supporting fiber that precedes a type I Collagen (mature) polymerization. In the tissue remodeling process, under conditions involving inflammatory events, the ECM turnover results in quantitative and qualitative changes of collagens. Therefore, damaged mature molecules (type I mature Collagen) are degraded and replaced by new ones (type III immature Collagen) to re-establish the homeostasis process [28]. However, considering an increased percentage of type III Collagen (immature) was found in the COVID-19 group, its complementary percentage of type I Collagen (mature) would be reduced.

In addition to the exacerbated inflammatory process observed in COVID-19 patients, it is worth reporting that ventilatory support can also cause lung injury due to repeated chest distensions and pressurizations, which, in turn, can intensify DAD [29,30].

### 3.2. Virally Mediated Fibrogenic Pathways

SARS-CoV-2 cell infection is dependent on ACE-2 receptors [31], and these receptors regulate the RAS [6]. SARS-CoV-2 preferentially infects type II pneumocytes, given this increased ACE-2 expression compared to type I pneumocytes [32]. However, this high affinity between S-Spike protein and ACE-2 can promote its downregulation and consequently accumulation of Ang II [33] with consequent activation of the ACE-AngII-AT1 axis that implies harmful effects ranging from vasoconstriction, inflammation, and fibrosis [29].

When evaluating the immunoexpression of ACE-2 in the present study, the COVID-19 group showed a tissue increase in this protein when compared to the CONTROL (*p* = *p* < 0.0001) and H1N1 (*p* = 0.0005) groups. The COVID-19 group comprises patients over 65 years of age exposed to mechanical ventilation. Baker et al. [34] demonstrated an increase in *ACE-2* gene expression and its protein immunoexpression in the alveolar epithelium of elderly and under mechanical ventilation patients. In addition, Wang et al. [35], in an in vitro study, identified recycling of ACE-2 back to the plasma membrane 14 h after endocytosis promoted by the S-Spike protein, demonstrating that ACE-2 would become suitable for its functions.

AngII-induced collagen expression is dependent on TGF-β [36]. Usually, when there is a higher activity of Ang II, there is an upregulation of TGF-β1. Upon binding to the Angiotensin II receptor (AT1R), Ang II activates the PI3K/AKT pathway that triggers the TGF-β1 activation and phosphorylation of R-SMAD proteins (SMAD 2/3), leading to excessive proliferation and differentiation of fibroblasts [37]. The ACE-AngII-AT1 axis may be activated in the COVID-19 patients, given that there was a higher immunoexpression of AKT-1 and TGF-β1 in those patients compared to the CONTROL group.

Another relevant finding is the decreased immunoexpression of Cav-1 in the COVID-19 group. The SARS-CoV-2 could infect the target cells by the endosomal route [15]. Recent reports show that this route depends on the lipid rafts, cellular membrane regions enriched by Cav-1 and ACE-2 receptors, endocytosed during the virus infection endosomal route [38]. In addition to the possible participation of the viral infection endosomal route, Cav-1 is also involved in the TGF-β pathway negative regulation. There are TGF-β receptors located into the lipid rafts, and when a physical interaction of Cav-1 and TGF-β receptors occurs, they are internalized. Once internalized, such receptors undergo degradation, culminating in an effective reduction in TGF-β signaling [17].

**Figure 4:**
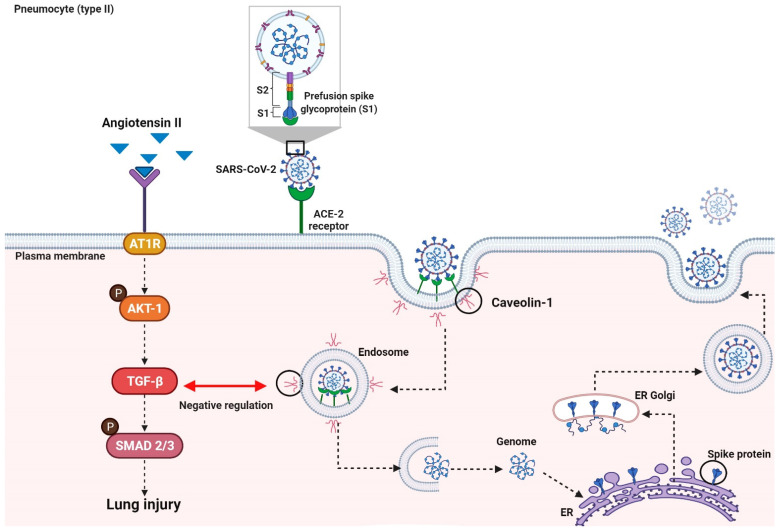
Virally Mediated Fibrogenic Pathways. The entry of SARS-CoV-2 into the host cell is dependent on ACE-2 receptors. The high-affinity binding between the S-Spike protein and ACE-2 receptors can trigger an imbalance of the RAS system, resulting in the accumulation of Ang II. When Ang II binds to the ATR1 receptor, it activates the PI3K/AKT pathway. TGF-β1 consequent activation triggers the SMAD2/3 pathway, which provides proliferation and differentiation of fibroblasts. Viral infection endosomal route involves Cav-1 and ACE-2 on the lipid rafts. Cav-1 is also involved in the negative regulation of the TGF-β pathway. The physical interaction of Cav-1 and TGF-β receptors on the lipid rafts results in the internalization and degradation of this receptor, culminating in a reduction in TGF-β signaling.

The TGF-β interaction with receptor types I and II a (TGF-βRI and TGF-βRII) triggers a cascade of intracellular phosphorylation involving SMAD1/2 and 4 transcription factors interactions. This SMAD1/2–4 complex is translocated to the nucleus and modulates the transcription of ECM genes involved in the fibrogenic process. [39,40]. Razani et al. [41], in their experimental study, showed that Cav-1 also promotes decreasing phosphorylation of SMAD2, interrupting its interaction with SMAD4 and, thus, avoiding the translocation of the complex to the nucleus (Figure 4).

The present work corroborates the literature, having seen the decreased immunoexpression of Cav-1 and the increased TGF-β1 in the COVID-19 samples compared to the CONTROL group, culminating in terminal lung fibrosis.

### 3.3. TGF-β Signaling in Pulmonary Fibrosis

TGF-β is a multifunctional cytokine that plays a crucial role in tissue repair after an injury. Three isoforms of TGF-β are found in mammals: TGF-β1, 2, and 3, and the signaling pathway of TGF-β1 are predominantly involved in the pathogenesis of fibrotic lung disease, because it induces fibroblast proliferation and differentiation [42]. There are reports that TGF-β1 induces lung fibrosis by activating SMAD-independent and SMAD-dependent pathways, increasing ECM and collagen deposition [43].

Due to the cytokine storm, an influx of macrophages, neutrophils, and T cells can occur at the site of infection, causing lung injury [44]. The presentation of antigens leads to the proliferation and differentiation of CD4+ T cells in subclasses such as Th2. Alternatively, activated M2 phenotype macrophages are induced by IL-4 [7]. An increased immunoexpression of Sphingosine-1 (M2 Macrophages) and IL-4 in the COVID-19 group was observed, suggesting that the Th2 pathway was activated.

Studies with patients committed with COVID-19 detected that elevated levels of IL-4 were associated with severe respiratory symptoms [45,46]. Furthermore, cytokines that stimulate the Th2 pathway can interfere with the T-helper 1 (Th1) response, as they suppress the activation of macrophages of the M1 phenotype. Th2 response, besides being inefficient in viral clearance [4], stimulates the production of cytokines such as TGF-β1, exacerbating the tissue remodeling process [7].

When evaluating the immunoexpression of α-SMA, CD44v6, and MMP-9, it was noted that they were elevated in the COVID-19 group. Fibroblasts can differentiate into myofibroblasts [47] that, due to their expression of α-SMA, can promote irreversible contractility leading to a spatial reorganization of collagen, a fundamental characteristic of fibrogenesis [42]. Myofibroblasts with high expression of α-SMA were found in pathological conditions characterized by fibrosis [48] and, due to their ability to express high levels of Collagen I (mature), ECM proteins, fibrogenic and inflammatory cytokines, they play a fundamental role in inflammation and deposition of connective tissue [49].

Hyaluronic acid (HA), also present in ECM, can increase the life of myofibroblasts, leading to the perpetuation of lung fibrosis [11]. CD44v6 is the central receptor for HA, a cell adhesion receptor that is upregulated after tissue damage and is implicated in many chronic inflammatory diseases [10]. The CD44v6/HA complex may lead to the PI3K/AKT pathway activation, which induces the reduction in fibroblast apoptosis [11]. These findings would also justify the increase in tissue immunoexpression of AKT-1 and CD44v6 in the COVID-19 samples.

**Figure 5:**
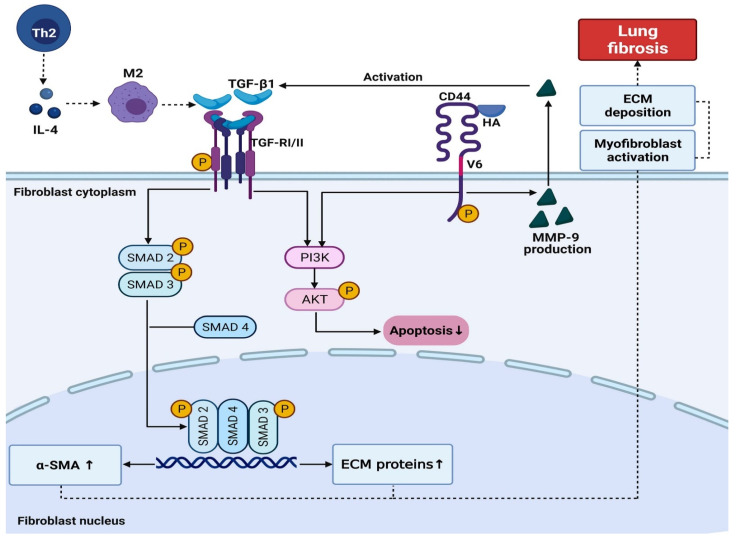
IL-4 mediates the functions of Th2 cells. IL-4 induces the alternative activation of M2 macrophages, which, in turn, secrete TGF-β1, stimulating fibroblast proliferation. The TGF-β type I and type II receptors (TGFRI and II) activate several intracellular signaling cascades, including the canonical SMAD2/3-4 and the noncanonical PI3K/AKT pathways. Once active, TGFRI triggers the complex SMAD2/3-4 that binds profibrotic genes and induces fibrogenic proteins expression such as α-SMA and Collagen I (mature), resulting in induction myofibroblast activation and ECM deposition. The PI3K/AKT pathway, on the other hand, increases the resistance of fibroblasts and the proliferation of myofibroblasts. Furthermore, once the CD44v6/HA complex is formed, the PI3K/AKT pathway leads to fibroblasts’ resistance to apoptosis. This CD44v6/HA interaction increases the production of MMP-9, which leads to the activation of TGF-β1.

Regarding MMP-9, several authors state a high expression of this molecule associated with lung injury processes [12,50]. In this scenario, resident pulmonary epithelial cells synthesize MMP-9 only when subjected to various forms of injury. Furthermore, the degradation of the extracellular matrix, the MMP-9 is responsible for activating TGF-β1 by binding the CD44v6 [50], see Figure 5.

### 3.4. Corticosteroid and Pulmonary Fibrosis

Among the medications used by COVID-19 patients were corticosteroids (*n* = 17 patients). Glucocorticoids are a class of drugs used as a first-line treatment in numerous inflammatory diseases [51]. During this pandemic period, studies have shown that corticosteroids could mitigate the effects of COVID-19-induced pneumonia by attenuating the inflammatory response, and their prolonged use could be beneficial in reducing the severity of post-COVID-19 complications [52].

The use of this pharmacological class to delays the progression of lung fibrosis is contradictory. It has been demonstrated, for example, that the use of prednisone slowed the progression of lung fibrosis in rats, and that the mechanism of action could be related to increased levels of Cav-1, reduction in tumor necrosis factor (TNF-α), TGF-β, and platelet-derived growth factor (PDGF) [53]. On the other hand, a study with a model of fibrogenesis attested that the profibrotic response was not reduced with dexamethasone, especially concerning the inhibition of TGF-β-dependent fibrogenesis [54]. Furthermore, Kuwano et al. and Bogliolo et al. mention that corticosteroid therapy is ineffective in preventing lung fibrosis progression [55,56].

In this present study, no statistical difference was observed when evaluating the use of this drug and the presence of pulmonary fibrosis (Table 3).

### 3.5. Limitation of the Study

As this is a retrospective study, in which samples were obtained from *post mortem* biopsies, the information from this study cannot reconstruct the events in a chronological evolution of the disease.

Since the number of samples is small, the results inferred in this study are considered preliminary, suggesting that future studies with larger samples be carried out.

## 4. Materials and Methods

### 4.1. Ethical Approval

The presented study was approved by the National Research Ethics Committee (Conselho Nacional de Ética em Pesquisa—CONEP), protocol number 3.944.734/2020, and 2.550.445/2018. The authors confirm that all methods were carried out following relevant guidelines and regulations. The families permitted the *post mortem* biopsy of the cases of COVID-19 and H1N1pdm09 and signed the informed consent forms.

### 4.2. Samples

COVID-19 group (*n* = 24): *Post mortem* lung samples from patients who died of SARS-CoV-2 infection. A minimally invasive lung biopsy was performed through a left anterior minithoracotomy with upper left lobe segment resection. The resected pieces were 3 × 3 cm. The time of acquisition after death was less than 2 h. Clinical data were obtained from medical records during hospitalization in the Intensive Care Unit (ICU) at the Marcelino Champagnat Hospital in Curitiba, Brazil. Testing for COVID-19 was performed by nasopharyngeal swabs taken during ICU hospitalization, as well as the performed Realtime Polymerase Chain Reaction (RT-qPCR). The viral genome amplification was performed with the Invitrogen SuperScript™III Platinum^®^ One-Step qRT-PCR Kit (Catalog number: 11732020, Waltham, MA, USA), were positive for SARS-CoV-2.

H1N1 group (*n* = 10): Lung samples from patients who died of H1N1pdm09 (*n* = 10) were obtained by minimally invasive lung *post mortem* biopsy (COVID-19 similar technique). The patients were tested for H1N1pdm09 through the qRT-PCR (COVID-19 similar technique).

CONTROL group (*n* = 11): Composed of necropsy lung samples from patients who died due to cardiovascular and neoplastic disease, not involving lung lesions.

### 4.3. Histological and Morphometric Analysis

All representative lung samples were formalin-fixed and paraffin-embedded (FFPE) and routinely processed to assess histopathological findings.

Histological sections were stained with Sirius Red (Direct Red: Aldrich Chemical Company Inc., Milwaukee, WI, USA) to evaluate Collagen I (mature) and III (immature) depositions. The slides were photographed at a magnification of 400× (high power field or HPF) under polarized light resulting in 20 images for each case. The Collagen I to III evaluation was performed using Image-Pro Plus 4.5 (Media Cybernetics, Rockville, MD, USA), where polarized areas (in red for mature Collagen I or in green for immature Collagen III) were identified. The values of the Collagen I (mature) and Collagen III (immature) analysis were expressed percentage per HPF.

### 4.4. Immunohistochemical Analysis

The immunohistochemistry assay was preceded by the preparation multisample paraffin tissue blocks, TMA (Tissue Microarray). The representative areas of the lung were previously demarcated and identified. Then, three cylindrical fragments measuring 0.3 cm in diameter were extracted from the original blocks (donor blocks) and compiled into new TMA blocks.

The immunohistochemistry technique was applied to identify the immunoexpression of ACE-2, AKT-1, Cav-1, CD44v6, IL-4, MMP-9, α-SMA, Sphingosine-1 (M2), and TGF-β1, as shown in Supplementary Table S2.

The technique recommended an overnight incubation protocol for primary antibodies. The secondary polymer (Mouse and Rabbit Specific HRP/DAB IHC Detection Kit-Micro-polymer, Abcam, ab236466, Cambridge, UK) was applied to the material at room temperature. The technique was revealed by adding the 2, 3, diamino-benzidine complex + hydrogen peroxide substrate. The positive and negative controls validated the reactions.

The slides of ACE-2, AKT-1, Cav-1, CD44v6, IL-4, MMP-9, α-SMA, and TGF-β1 were scanned with Axio Scan.Z1 Scanner (ZEISS, Jena, Germany), and then the software ZEN Blue Edition (ZEISS, Jena, Germany) was utilized for the generation of 30 HPF (COVID-19 group) and 20 HPF (H1N1 and CONTROL groups), randomly. The analysis was blind, once the software randomly generated the images, with no investigator’s interference. The immunopositivity areas were measured by the Image-Pro Plus 4.5. Subsequently, these areas were converted into percentages.

The slides immunostained by Sphingosine-1 were observed under an optical microscope and analyzed in ten HPF, using the scoring method known as Allred Score. This method evaluates the proportion and intensity of immunopositivity of M2 macrophages and type II pneumocytes. The semiquantitative analysis was obtained by summing two scores (proportion and intensity of positivity), ranging from 0 to 8. The proportion score was subdivided according to the percentage of cellular immunoexpression, in which cell score could be score 0–0% stained cells, score 1: < 1%, score 2: 1–10%, score 3: 11–33%, score 4: 34–66% and score 5: >66%. While the intensity of positivity was evaluated: negative: score 0, weak: score 1, moderate: score 2, and strong: score 3.

### 4.5. Statistical Analyses

The comparison of quantitative variables between two groups was performed using the non-parametric Mann Whitney test. For demographic variables, the T-Student parametric test was used. For categorical variables, Fisher’s exact test was used. Values of *p* < 0.05 indicated statistical significance. Data were analyzed using JMP(™) Pro 14.0.0 software (SAS 483 Institute, Cary, NC, USA).

## 5. Conclusions

Altogether, the results suggest the participation of TGF-β pathways in the development process of terminal septal alveolar fibrosis. Considering the prevalence and characteristics of the COVID-19 pandemic, it is very likely that the SARS-CoV-2 will become an endemic virus. The vaccines have proven themselves as great agents in the containment of the disease, but on the other hand, at this moment, there is still serious concern with post-COVID-19 sequelae, especially those concerning pulmonary fibrosis. In this context, therapy such as the anti-TGF-β monoclonal antibody could be evaluated to minimize the lung damage caused by ARDS and its consequent repair by the fibrogenic process.

## Figures and Tables

**Table 1:** Comparison between COVID-19, H1N1, and basal CONTROL groups according to clinical findings and pathology features.

Data	CONTROL (*n* = 11)	COVID-19 (*n* = 24)	H1N1 (*n* = 10)
***Gender ^a^***	Male (8) 72.7%	Male (15) 62.5%	Male (8) 80.0%
	Female (3) 27.3%	Female (9) 37.5%	Female (2) 20.0%
	0.709 *		0.437 **
***Age (years) ^1,b^***	42.31 ± 4.4	71.96 ± 12.5	43.5 ± 14.0
	<0.001 *		<0.001 **
***Time from hospitalization to death (days) ^1,b,c^***	7.6/13.1	15.87 ± 10.2	4.70 ± 6.13
	0.003 *		0.003 **
***Mechanical ventilation ^1,b^***	-----	12.0 ± 9.2	4.7 ± 6.13
	-----		0.028 **
***Previous pulmonary diseases***	-----	Bronchial Asthma (4/24) Interstitial Pulmonary Fibrosis (1/24)	-----
***Histological pattern of DAD***	Normal septum	Interstitial pneumonitis with scarce septal neutrophils, hyaline membrane, type II pneumocyte hyperplasia, fibrosis, and micro thrombosis	Interstitial pneumonitis with high septal neutrophils infiltration and no micro thrombosis
***Computed tomography chest at admission***	-----	“Opacities with ground-glass attenuation,” suggestive of viral pulmonary infection (24/24); Interstitial Pulmonary Fibrosis (1/24); Bronchial thickening (3/24); Bilateral pleural thickening (2/24); Pleural effusion (4/24); Parasseptal emphysema (2/24); Pulmonary consolidation (1/24);	-----
***Anti-inflammatory drugs***	-----	Dexamethasone 6 mg/day (12/24); Hydrocortisone 100 mg/day (1/24); Hydrocortisone 200 mg/day (3/24); Methylprednisolone 125 mg/day (2/24); Prednisone 60 mg/day (1/24); Prednisone 10 mg/day (1/24);	-----

Subtitle: ^1^ Average/Straight deviation (Min-Max); * = *p*-values obtained were compared between COVID-19 versus CONTROL group. ** = *p*-values obtained were compared between COVID-19 and H1N1 group; *^a^ p*-values were performed using the Fisher’s test (*p* < 0.05); *^b^ p*-values were performed using the parametric Student’s test (*p* < 0.05); *^c^ p*-values were performed using the non-parametric Mann Whitney test (*p* < 0.05).

**Table 2:** Histopathological findings and morphometric evaluation of Collagen I (mature) and III (immature).

Data	Category	CONTROL (*n* = 11)	COVID-19 (*n* = 24)	H1N1 (*n* = 10)
***DAD ^a^***	Absent	11 (100.0%)	0 (0%)	0 (0%)
	Initial	0 (0%)	10 (41.7%)	5 (50.0%)
	Established	0 (0%)	14 (58.3%)	5 (50.0%)
		<0.001 *		0.718 **
***Hyaline membrane ^a^***	Absent	11 (100%)	3 (12.5%)	6 (60.0%)
	Present	0 (0%)	21 (87.5%)	4 (40.0%)
		<0.001 *		0.009 **
***Type II pneumocyte hyperplasia ^a^***	Absent	6 (54.5%)	1 (4.2%)	0 (0%)
	Present	5 (45.5%)	23 (95.8%)	10 (100.0%)
		0.0002 *		1.000 **
***Fibrosis***	Absent	11 (100.0%)	2 (8.3%)	4 (40.0%)
	Present	0 (0%)	22 (91.7%)	6 (60.0%)
		<0.001 *		0.048 **
***Collagen I (mature) ^b^***	-	95.47 (60.32–99.51) ^1^	68.53 (6.93–99.38) ^1^	97.21 (70.88–99.51) ^1^
		0.0015 *		0.0006 **
***Collagen III (immature) ^b^***	-	4.52 (0.49–39.68) ^1^	31.47 (0.62–93.06) ^1^	18.10 (8.95–44.35) ^1^
		0.0015 *		0.2863 **

Subtitle: ^1^ Median (Min-Max); * = *p*-values obtained were compared between COVID-19 versus CONTROL. ** = *p*-values obtained were compared between COVID-19 and H1N1 group; *^a^ p*-values were performed using the non-parametric Fisher’s test (*p* < 0.05); *^b^ p*-values were performed using the non-parametric Mann Whitney test (*p* < 0.05).

**Table 3:** Relation of Corticosteroid use and PF development.

Fibrosis	Corticosteroid Use	No Corticosteroid Use
Absent	0 (0%)	2 (28.6%)
Present	17 (100.0%)	5 (50.0%)
	0.718	

*p*-values were performed using the Fisher’s test (*p* < 0.05).

## Supplementary Materials

The following are available online at https://www.mdpi.com/article/10.3390/ijms23010168/s1.
