# Supplementary material for: COVID-19: Immunohistochemical Analysis of TGF-β Signaling Pathways in Pulmonary Fibrosis
Source: Int J Mol Sci. 2021 Dec 24;23(1):168. doi: 10.3390/ijms23010168 (PMC8745764; doi:10.3390/ijms23010168)
Supplement: Supplementary file 1 [file ijms-23-00168-s001.zip › ijms-1503793-supplementary.pdf]

Supplementary Table S1. Comparison between basal CONTROL, COVID-19 and H1N1 Groups according to immunohistochemical findings.

Data	CONTROL (N=11)	COVID-19 (N = 24)	H1N1 (N = 10)
Anti-ACE-2 [1]	2.14 (0.46-5.16)	9.76 (3.1-34.95)	3.07 (1.51-9.03)
	**<0.0001***		**0.0005****
Anti-AKT-1 [1]	0.31 (0.12-2.49)	1.39 (0.1-8.34)	0.85 (0.15-5.54)
	**0.022***		0.249**
Anti-Caveolin-1 [1,a]	24.14 (16.87-28.76)	4.34 (0.23-16.34)	4.84 (1.6-12.46)
	**<0.0001***		0.668**
Anti-CD44v6 [1]	0.27 (0.09-2.2)	7.89 (2.42-22.22)	0.09 (0.003-0.18)
	**<0.0001***		**<0.0001****
Anti-IL-4 [1]	2.11 (0.23-11.24)	17.49 (1.02-32.4)	1.35 (0.36-3.41)
	**<0.0001***		**<0.0001****
Anti-MMP-9 [1]	2.33 (0.46-8.62)	4.95 (1.86-18.42)	6.91 (3.33-22.8)
	**0.004***		0.609**
Anti-α-SMA [1]	0.39 (0.02-2.46)	7.46 (2.57-37.15)	0.57 (0.07-1.75)
	**<0.0001***		**<0.0001***
Anti-Sphingosine-1 [1]	4 (3-5)	6 (5-7)	5 (4-6)
	**<0.0001***		0.063**
Anti-TGF-β1 [1]	1.13 (0.4-7.04)	2.97 (0.69-21.91)	3.18 (1.16-5.06)
	**0.0315***		0.677**

Subtitle:[1] Median (Min-Max); [a] n=23;* = *p*-values obtained were compared between COVID-19 versus COTROL. ** = *p*-values obtained were compared between COVID-19 and H1N1 group; *p*-values were performed using the non-parametric Mann Whitney test (p <0,05).

Supplementary Table S2: Resource table

Antibody	Type	Clone/Code	Dilution	Source	Species Reactivity	RRID
Anti-ACE-2	Polyclonal/Rabbit	Ab272690	1:50	Abcam	Human[1]	*
Anti-AKT-1	Monoclonal/Rabbit	Y89	1:200	Abcam	Human[2]	*
Anti-Caveolin-1	Monoclonal/Rabbit	EP353	1:200	BioSB	Human[3]	*
Anti-CD44v6	Monoclonal/Mouse	VFF-7	1:200	Novocastra	Human[4]	*
Anti-IL-4	Polyclonal/Rabbit	PA5-25165	1:200	Thermo Fisher	Human, Mouse[5]	AB_2542665
Anti-MMP-9	Monoclonal/Rabbit	EP1254	1:200	Abcam	Human, Rat[6]	AB_1267245
Anti-α-SMA	Polyclonal/Rabbit	Ab5694	1:600	Abcam	Human, Mouse[7]	*
Anti-Sphingosine-1	Polyclonal/Rabbit	Ab71700	1:200	Abcam	Human, Rat, Mouse[8]	AB_1270891
Anti-TGF-β1	Polyclonal/Rabbit	E11262	1:200	Spring	Human[9]	AB_11219319

[1] https://www.abcam.com/ace2-antibody-ab272690.html
[2] https://www.abcam.com/akt3--akt2--akt1-antibody-y89-ab32505.html
[3] https://www.biosb.com/biosb-products/caveolin-1-antibody-rmab-ep353/
[4] https://issuu.com/leicabiosystems/docs/2013_ihc_and_ish_product_catalog_ro/119
[5] https://www.thermofisher.com/antibody/product/IL-4-Antibody-Polyclonal/PA5-25165
[6] https://www.abcam.com/mmp9-antibody-ep1254-ab76003.html
[7] https://www.abcam.com/alpha-smooth-muscle-actin-antibody-ab5694.html
[8] https://www.abcam.com/sphk1-antibody-ab71700.html
[9] https://issuu.com/fermelobiotec/docs/catalogo_spring_bioscience
***** There is no record.
